# The National Alcohol Survey of Households in Trinidad and Tobago (NASHTT): willingness to support changes in policy, laws and regulations

**DOI:** 10.1186/s12889-018-6111-4

**Published:** 2018-10-25

**Authors:** Rohan G. Maharaj, Terence Babwah, M. Shastri Motilal, Paula Nunes, Rachel Brathwaite, George Legall, Sandra D. Reid

**Affiliations:** 1 The Unit of Public Health and Primary Care, Department of Paraclinical Sciences, The University of the West Indies, St. Augustine, Trinidad; 2 Alcohol Policy Advisor, The Healthy Caribbean Coalition, Bridgetown, Barbados; 3 The Unit of Psychiatry, Department of Medicine, The University of the West Indies, St. Augustine, Trinidad

**Keywords:** Alcohol drinking, Trinidad, Tobago, Public opinion, Policy

## Abstract

**Background:**

Over 60% of households (HHs) in Trinidad and Tobago (T&T) consume alcohol. These HHs were more likely to report illnesses, relationship problems, and behavioral problems with children. This study set out to determine what proportion of HHs were willing to support changes in specific policies, laws and regulations in a national alcohol campaign.

**Methods:**

A cross-sectional convenience sample of HHs were surveyed from a random sample of enumeration districts (ED) in T&T. An interviewer-applied, field pre-tested de novo questionnaire had 5 domains and was developed over 1 1/2 years after an extensive literature review and consultation. Many of the WHO ‘best buys’ recommendations were included.

**Results:**

One thousand six hundred ninety-five HHs (from 53 ED) responded from a total of 1837 HHs approached (response rate 92%). In a national campaign the following proportions of HHs would support: setting the legal age for drinking at 21 years (82.4%); restricting or banning alcohol advertising on TV and other media (73.1% and 54.4% respectively); banning all alcohol advertising at sports and cultural events (64.8%); banning radio stations playing songs with reference to alcohol use (71.3%); holding sellers of alcohol responsible for the amount of alcohol sold (79.5%); advocating that proof of age to be shown by persons buying alcohol (87.4%); placing more prominent warning labels on products displaying alcohol content (87.2%); placing more prominent warning labels on products showing harmful effects (88.5%); increasing taxes on alcohol sales (87.7%). Less than 50% of HH supported restrictions in density of outlets and reduction in opening times for alcohol outlets.

**Conclusions:**

Many HHs in T&T are willing to support changes in policies around alcohol, including many of the policies shown by the WHO to be effective in reducing the harmful consumption of alcohol.

## Background

Alcohol is associated with adverse medical and social conditions, both acute and chronic [1]. Indeed, alcohol presents an ongoing Caribbean public health problem. The Global Student Health Survey reported that in studies between 2003 and 2010, 45% of youth aged 13–15 years in the English speaking Caribbean (ESC) self-reported alcohol use in the past 30 days [2]. More disturbing was the report that 22% of this group reported drinking so much alcohol that they staggered, vomited, or developed slurred speech at least once in their life [2]. Among adults 15–64 years old, in Trinidad and Tobago (T&T), 40.4% consumed alcohol in the past 30 days, 50.6% males and 30.9% females. This increases to 50.2% among the 25–34 year age sub-group, for both sexes [3]. The percentage of males who engaged in heavy episodic drinking (HED), ranged between 22% in Barbados and 33.9% in Trinidad and Tobago and between 9.7 and 16.8% for females in these islands respectively [3, 4]. Among the countries in the Americas in 2010, 72.9% of male youth in T&T admit to HED, the highest in the region [5].

In T&T, noncommunicable diseases (NCDs) account for 80% of overall mortality [6]. Alcohol use is recognized as a major contributor to NCDs including hypertension, stroke, cancer, and liver disease and is a major contributor to violence, including domestic violence, and injury among peoples of Latin America and the Caribbean (LAC). In 2002, alcohol was responsible for nearly 10% of all Disability Adjusted Life Years (DALY) lost in the LAC, compared to the global figure of 4.4% [6]. Alcohol contributed to 20–50% of road traffic fatalities in the LAC, and 50.5% of the alcohol-attributable deaths in the Americas in 2002 were due to injuries [7]. In 2005, adult per capita consumption of alcohol in the Americas was 8.7 L per year—higher than the world average of 6.1 L [7].

The role of alcohol as a contributor to disease, notably NCDs has been recognized by Caribbean governments. In 1999, The Caribbean Cooperation in Health Phase II (CCH II) identified the strengthening of alcohol prevention and control programmes as a priority issue in the context of the prevention of mental health disorders [8]. And in 2007, the Declaration of Port of Spain acknowledged alcohol as a causal risk factor for NCDs [9].

Despite these statistics and declarations there is ambivalence towards alcohol in the media and the general population. All the countries in the archipelago and on the mainland, have some restrictions on alcohol notably excise duty and taxes, regulations on underage sales, and drunk driving, but only The Bahamas and Jamaica are reported to have any restrictions on advertising and marketing [10]. Alcohol can be sold by many establishments in Trinidad and Tobago, for example, on an ‘any day, any time’ basis, and many petrol stations also sell alcohol. Many politicians are involved in protecting the local and regional alcohol production and trade against global interests [11]. See Table 1 for a summary of laws and regulations on alcohol in T&T [12].

**Table 1:** Alcohol related policy in Trinidad and Tobago [12]

National Policies and Government Support; National Monitoring Systems	Present?
Written national policy (adopted/revised)/National Action Plan	No^a^
National government support for community action	Yes
National monitoring system(s)	Yes
Taxes and National Maximum Blood Alcohol Concentration (BAC)	
Excise tax on beer/ wine/ spirits	Yes (all three)
National maximum legal blood alcohol concentration (BAC) when driving a vehicle (general/young/professional)	0.08% (for all 3)
Minimum Age interactions	
National legal minimum age for off-premise sales of alcoholic beverages (beer, wine, spirits)	18 years
National legal minimum age for on-premise sales of alcoholic beverages (beer, wine, spirits)	18 years
Minimum drinking age	None
Restrictions for on−/off-premise sales of alcoholic beverages	
Time (hours and days) / location (places and density)	No, No/ Yes, No
Specific events / intoxicated persons / petrol stations	Yes/ Yes/ No
Advertising, Marketing and Labeling	
Legally binding regulations on alcohol advertising / product placement	No
Legally binding regulations on alcohol sponsorship / sales promotion	No
Legally requiring health labels on alcohol advertisements/containers,	No

^a^A draft policy has been created but it has not been put forward for national discussion, disseminated or accepted by the Cabinet of the government

There is a strong historical connection to alcohol, notably rum, in the Caribbean. These connections are over 300 years old [13–15]. Many of the globally recognized icons of Caribbean excellence are alcohol based: Angostura (Trinidad), Demerara Rums (Guyana), Appleton (Jamaica), and Mount Gay (Barbados). Tourism remains the major source of revenue for most islands in the region and many fear that placing restrictions on alcohol will harm this trade. However, there is a public health price.

Before policies and strategies can be developed and implemented to temper harmful alcohol use, an assessment of the population’s desire for change needs to be conducted.

An aim of the NASHTT was to determine the changes to alcohol related laws, regulations, advertisements and other policies that would be acceptable to households in Trinidad and Tobago and what proportion of HHs would be willing to support these changes? Also we set out to determine whether there were significant differences in support between HH where alcohol was consumed versus those where it was not; and HH where there was HED versus those where there was no HED?

## Methods

### Design

A cross-sectional convenience sample of households (HH) was surveyed from a random selection of enumeration districts in Trinidad and Tobago, the questionnaire was applied by the interviewer in a face-to-face session.

### Instrument

A de novo questionnaire was developed after a literature review and formal consultation with family physicians, a psychiatrist, a sociologist, a statistician and public health specialists. Documents such as the draft Ministry of Health of Trinidad and Tobago’s National Policy on Alcohol [16] and Babor’s *Alcohol: No Ordinary Commodity* [1] provided background information for section 4 of the instrument (See below). The survey instrument had five [5] domains with 50 items altogether and was developed over the period January 2012–March 2013. The 5 domains were:

1. The HH’s use of alcohol
2. The social, medical and psychological impact of alcohol use on the respondent’s immediate HH, family members or relatives, friends or co-workers. The results of the survey findings of these first 2 domains have been published [17].
3. The HH’s impression of the impact of the alcohol trade on their immediate residential environment.
4. The HH’s willingness to support changes in national alcohol policies in Trinidad and Tobago.
5. HH’s demographics

The instrument was pre-tested on over 40 participants and feedback incorporated.

### The interviewers

With assistance from the Central Statistical Office (CSO) a group of experienced enumerators were identified. These were mature individuals who had worked previously in conducting numerous HH surveys including a recently concluded national census. A training manual and field manual were created and the interviewers underwent a half day training session.

### Sample size

The sample of the NASHTT was based on the Continuous Sample Survey of the Population (CSSP) conducted by the CSO of the Government of Trinidad and Tobago biannually. This CSSP reaches approximately 3200 HHs or 1.5% of the national HHs.

### Selection of HH

The CSO provided a list of national Enumeration Districts (EDs) and from these a random sample of EDs was taken, Tobago included. There are 2824 EDs in T&T. EDs represent HHs of similar economic status and may contain between 250 and 600 HHs. We selected 72 EDs, using a random number generator from Excel. The 72 maps for each ED were purchased from CSO. Using the ED maps, interviewers planned to interview about 40–50 HHs per ED, hence achieve the sample of the CSSP. A sampling interval of 3–6 was used for each ED, depending on the size of the EDs. Larger EDs (HHs greater than 300) used a sampling interval of 6, Medium EDs (HHs between 250 and 300) a sampling interval of 5 and EDs between 200 and 250 HHs, a sampling interval of 3–4. Enumerators started the sampling at a fixed point set by the CSO in each ED and applied the sampling interval for that ED.

Because of limited financial resources, return visits to HH was not possible. As such, if one HH refused to participate or was ineligible to participate, successive HH were approached, immediately next to the HH that refused, until a response was received. After this the sampling interval for that ED was reapplied. In the case that the HH on the map is found to be a condominium or apartment building, the same interval strategy was used, counting each condominium or apartment as an individual household.

### Selection of respondent

Persons eligible to answer the questionnaire were in the order of preference: 1. “Household head” or whosoever the person answering the door identified as household head; 2. Any person over the age of 18 years who was knowledgeable of the HH and willing to participate. Only one [1] questionnaire per household was allowed, but more than one person in the HH could have contributed answers.

### Non-response rate

Non-consenting HHs were recorded to determine the non-response rate.

### Consent process

Once a possible respondent was identified information on the study was shared. The interviewer read out the preamble to the questionnaire which provided extensive information on the purpose of the study and what would be required of participants. Agreeing participants completed and signed the accompanying consent form. Participants were given their signed consent form and signed a separate consent sheet for researchers’ records.

### Statistical analysis

This was done using SPSS v 20, IBM, Chicago. Demographic data and independent variables were analysed using simple frequencies. Cross tabulation was done between the dependent variables (HH where persons consume alcohol OR HH where no alcohol is consumed) AND each of the proposed policies.

Cross tabulation was done between the dependent variables (HH by reported Major Ethnicity (Afro-Trinidadian/Indo-Trinidadian/Mixed Ethnicity/Other), Highest achieved Education (Primary (7 years of education) or Lower/Secondary (12–14 years of education)/ Post Secondary (17 years and beyond) and reported Income Level (Low/ Low Middle/ High Middle/ High) of the HH AND each of the proposed policies. Chi square and associated *p* values were then obtained. The level of significance was set at alpha = 0.5.

Cross tabulation was done between the dependent variable ‘heavy episodic drinking (HED)’ or ‘no HED’ by HH and each of the proposed campaigns. Chi square and associated *p* values were then obtained. The level of significance was set at alpha = 0.5.

Binary logistic regression was conducted with the change in policy as the dependent variable, and the demographic as the covariate. The following were used as the comparator variable: Afro-Trinidadian for ethnicity, Low income for income and primary/lower for education). ORs, CIs and *p-*values were calculated.

## Results

Fifty-three EDs accounting for 1.8% of national EDs, were surveyed. Of 1837 households approached, 1695 responded (response rate 92%). In 81.5% of the households (HHs) the head of the HH was the main respondent. In total the 1695 HH represented 5525 adults (2734 men and 2791 women) and 1553 persons under 18 yrs. HH reported the predominant ethnicity as African (41.4%), East Indian (29.4%) and mixed (28.1%). At least one person was employed fulltime in 80.4% of the HHs and the self reported income were low (31%), low middle (53%), upper middle (15%) and high (1%) income categories respectively. The most common type of dwelling was privately owned housing (80.8%) and most dwellings were either brick or concrete structures (76.9%). The highest level of schooling completed by the head of the HH was primary (25.8%), secondary (40.5%) and tertiary (23.1%) level respectively. With regards to monthly income brackets (TT dollars), HHs reported distribution was $2000–$4999 (28.7%), $5000–$7999 (19.7%), $8000–$9999 (15.2%) and $10,000–19,999 (19.5%).

### What would your HH support in a national campaign?

#### Drinking age

In a national campaign over 80% of HH would support setting the legal age for drinking at 21 years and advocating for proof of age to be shown by persons purchasing alcohol. Almost 80% would support holding the seller responsible for alcohol sales.

#### Breathalyzer

Nine out of 10 respondents would support stricter enforcement of the breathalyzer, and an increased public education campaign. See Table 2.

**Table 2:** Test of association between HH where alcohol is used and those where alcohol is not used and willingness to support national alcohol campaigns

National Campaign (Willingness to support)	HH not using alcohol *N* = 638 (%)	HH using alcohol *N* = 1055 (%)	Chi square, *p* value
Breathalyzer/driving			
Stricter enforcement of breathalyser	582 (91.2)	936 (88.7)	2.4, 0.12
Increase fines for drunk driving	567 (88.8)	900 (85.3)	3.9, 0.048
Taxation			
Increase taxes on alcohol sales	580 (90.9)	890 (84.4)	14.3, < 0.001
Public education			
Increase public education campaign	582 (91.2)	950 (90.0)	0.52, 0.47
Advertising and media			
Restrict alcohol advertising on TV etc	470 (73.7)	765 (72.5)	0.2, 0.64
Ban all alcohol advertising on TV etc	389 (61.0)	504 (47.8)	27.3, < 0.001
Ban all alcohol advertising at sports, cultural events	428 (67.1)	659 (62.5)	3.5, 0.06
Ban radio stations playing songs with reference to alcohol use	485 (76.0)	702 (66.5)	16.6, < 0.001
Delink consumption of alcohol with social success and sex	417 (65.4)	632 (59.9)	4.8, 0.03
Delink consumption of alcohol with driving and physical performance	443 (69.4)	731 (69.3)	0.0, 0.99
Adolescent issues			
Set legal age limit for drinking at 21 yrs	514 (80.6)	888 (84.2)	3.3, 0.07
Hold sellers of alcohol responsible for the amount of alcohol sold	527 (82.6)	806 (76.4)	8.8, 0.003
Advocate proof of age to be shown by persons buying alcohol	557 (87.3)	922 (87.4)	0.00, 1
Labelling and retail sales			
More prominent warning labels on products displaying alcohol content	566 (88.7)	904 (85.7)	2.9, 0.09
More prominent warning labels on products showing harmful effects	575 (90.1)	917 (86.9)	3.6, 0.06
Reduce availability			
Reduce opening hours of bars and rum shops	241 (37.7)	396 (37.5)	0.61, 0.74
Would like to see fewer bars and rum shops operating in your community	177 (19.8)	127 (12.1)	18.9, < 0.001

#### Advertising

The majority, 50–73%, of households supported changes in the advertising and delinking advertising with social success, sex, driving and physical performance on TV and other media, and banning songs on the radio that have a reference to alcohol use. See Table 2.

#### The nature of advertising

62.7% of HH would support a campaign to delink consumption of alcohol with social success and sex and 69.4% would support a campaign to delink consumption of alcohol with driving and physical performance. See Table 2

#### Retail sales

Regarding retail sales of alcohol more than 75% of HH would support a national campaign holding sellers of alcohol responsible for the amount of alcohol sold (to a particular costumer on any one occasion), advocating that proof of age to be shown by persons buying alcohol; placing more prominent warning labels on products displaying alcohol content; placing more prominent warning labels on products showing harmful effects. See Table 2 for further description.

#### Density of alcohol outlets

87.1% of HH reported having more than one outlet for retail alcohol sales within walking distance of their residence (1–2 km) and 31.9% reported having more than three. Between 5 and 15% of HH reported being annoyed by the number of bars in their neighborhood (10%), wishing to see fewer bars in their neighborhood (15%), being disturbed by noise from the bars (8%) and being disturbed by patrons of the bars (8%). In a national campaign 37.6% of HH would support reducing the opening hours of bars and rum shops.

#### Taxation

In a national campaign over 80% of HH would support increasing taxes on alcohol sales.

### Comparison of willingness to support national campaigns between HH where alcohol is used and those where alcohol is not used

Table 2 provides a comparison of the willingness of HH to support a national campaign based on whether alcohol is used in that HH or not. Generally there was wide support for the proposed policies regardless of whether alcohol was consumed in that HH or not. There was less support by HHs in which alcohol was consumed for: banning all alcohol advertising on TV and media, banning radio stations from playing songs with reference to alcohol, holding sellers of alcohol responsible for the amount sold, increasing taxes and increasing fines for drunk driving (*p* < 0.05).

We conducted a regression analysis on the statistically significant elements and found that only one campaign remained significant depending on whether the HH used alcohol or not. The odds of favouring a ban on all alcohol advertising on TV is 1.53 times higher among households in which no alcohol is consumed than among households in which alcohol is consumed.

### What would your HH support in a national alcohol campaign by characteristics of HH?

Table 3 provides the results of the analysis of the variety of policies supported by the HH, depending on the characteristics of the HH.

**Table 3:** Willingness to support national campaigns by demographics

Which of the following changes do you believe members of this household would support in a national campaign?	Unadjusted Odds Ratio (95% confidence interval)		
	Ethnicity^a^ East Indian vs. African; Mixed vs. African	Education level^b,c^ Secondary vs. Primary/lower; Post- secondary vs. Primary/lower	Income Level^d^ Low middle vs. Low; Upper middle vs. Low; High vs. Low
Set the legal age for drinking at 21 years	**2.364 (1.675–3.336)** 1.282 (0.949–1.732)	**1.455 (1.058–2.002)** 0.855 (0.633–1.155)	1.498 (0.885–2.534) **2.109 (1.262–3.525)** 1.704 (0.952–3.050)
Restricting alcohol advertisements on TV/radio/newspapers/cinema	**2.305 (1.738–3.057)** 1.206 (0.934–1.557)	**1.391 (1.077–1.797)** **1.476 (1.122–1.942)**	1.094 (0.679–1.764) **1.751 (1.097–2.793)** **2.291 (1.330–3.945)**
Ban of all alcohol advertisements on TV/radio/newspapers/cinema	**2.537 (1.992–3.230)** 0.959 (0.759–1.212)	**1.381 (1.100–1.732)** **1.323 (1.042–1.681)**	0.969 (0.613–1.531) 1.146 (0.735–1.785) 1.404 (0.856–2.304)
Ban of all alcohol advertisements at cultural or sporting events	**1.466 (1.141–1.883)** **0.744 (0.586–0.945)**	1.049 (0.829–1.328) 1.006 (0.785–1.289)	**2.236 (1.406–3.555)** **2.562 (1.634–4.017)** **2.935 (1.767–4.875)**
Delinking the consumption of alcohol with social or sexual success	1.201 (0.942–1.530) **0.742 (0.585–0.942)**	1.021 (0.810–1.286) 1.246 (0.972–1.598)	**1.879 (1.184–2.981)** **2.097 (1.341–3.281)** **3.032 (1.823–5.042)**
Delinking the consumption of alcohol with driving or physical performance	0.941 (0.728–1.216) **0.649 (0.505–0.833)**	0.834 (0.653–1.065) 0.791 (0.612–1.022)	**2.633 (1.654–4.190)** **2.912 (1.858–4.564)** **3.421 (2.046–5.719)**
Ban of radio stations playing songs which reference alcohol use	**2.147 (1.635–2.819)** 1.008 (0.788–1.290)	0.959 (0.750–1.225) 1.057 (0.813–1.373)	**1.882 (1.182–2.997)** **2.074 (1.323–3.253)** **2.209 (1.324–3.683)**
Stricter and more intensive enforcement of breathalyzer	**1.520 (1.022–2.261)** 1.209 (0.827–1.766)	0.706 (0.494–1.010) 0.953 (0.636–1.429)	**2.248 (1.258–4.017)** **2.821 (1.611–4.940)** **2.651 (1.361–5.163)**
Holding sellers of alcohol responsible for the amount of alcohol they sell to patrons.	**1.543 (1.151–2.070)** 1.100 (0.833–1.455)	0.819 (0.625–1.073) 1.052 (0.781–1.418)	**2.494 (1.548–4.020)** **3.076 (1.939–4.878)** **2.926 (1.715–4.992)**
Advocating for proof of age to be shown before alcohol is sold to a buyer	**1.524 (1.046–2.221)** 0.875 (0.626–1.224)	**0.546 (0.394–0.757)** 0.892 (0.610–1.304)	**2.952 (1.738–5.014)** **3.343 (2.015–5.545)** **3.692 (1.988–6.858)**
More prominent warning labels on products displaying the alcohol concentration	1.370 (0.951–1.974) 0.902 (0.644–1.261)	**0.546 (0.395–0.757)** 0.790 (0.547–1.140)	**3.171 (1.860–5.405)** **3.305 (1.993–5.479)** **2.431 (1.360–4.347)**
More prominent warning labels on products displaying the harmful effects of alcohol	1.463 (0.994–2.153) 0.888 (0.627–1.257)	**0.567 (0.401–0.802)** **0.645 (0.444–0.938)**	**3.335 (1.921–5.791)** **3.093 (1.844–5.188)** **2.790 (1.515–5.138)**
Increased taxation on alcohol	**1.544 (1.068–2.234)** 0.931 (0.668–1.298)	**0.714 (0.517–0.987)** 1.004 (0.695–1.450)	**2.280 (1.328–3.917)** **2.692 (1.600–4.529)** **2.977 (1.589–5.576)**
Increase the public education campaigns on responsible alcohol use in all settings including in schools	1.227 (0.799–1.885) 0.701 (0.479–1.027)	**0.550 (0.377–0.804)** 0.692 (0.457–1.050)	**2.799 (1.547–5.064)** **2.985 (1.701–5.239)** **2.930 (1.487–5.775)**
Increased fines for drunk driving	1.390 (0.979–1.971) 1.170 (0.832–1.645)	**0.643 (0.465–0.889)** 0.830 (0.580–1.188)	**2.490 (1.456–4.260)** **2.435 (1.462–4.055)** **5.032 (2.544–9.955)**

Odds ratios in bold show significant associations. Confidence intervals are either greater than or less than 1

^a^Comparisons in Ethnicity for East Indian vs. African and Mixed vs. African

^b^Education levels- Primary- 7 years of education; Secondary- 12-14 years of education; Post-secondary- 17 years and beyond

^c^Comparisons in Educations level for Seconday vs.Primary/lower and Post- secondary vs. Primary/lower

^d^Comparisons in Income level for Low middle vs. Low; Upper middle vs. Low and High vs. Low

#### Ethnicity of HH

Compared to African HHs, East Indian HHs were more likely to support raising the legal age to 21 yrs., restricting or banning all advertisements, banning radio songs which reference alcohol use, stricter breathalyzer enforcement, holding alcohols sellers responsible for the amount of alcohol they sell, advocating for proof of age before alcohol is sold and increased taxation.

#### Highest education level achieved by head of HH

HH where the highest level of education achieved by the head of the HH was to the secondary level HHs were more likely to support raising the legal age to 21 yrs. and restricting or banning all advertisements in media. However compared to Primary/Low education, Secondary level HHs were less likely to support advocating proof of age, more prominent warnings on products, increases taxation, increasing education campaigns and increasing fines.

#### Self-reported income level of HH

Generally higher income categories when compared to low income categories were associated with support for all the changes except regarding the ban on all advertisements.

### Heavy episodic drinking (HED) within HH and the support of policy

Table 4 illustrates that in both HH where HED occurred and HH where no HED occurred there was no difference in support for most of the proposed policies and regulation changes, Significant differences occurred however, with less support among HH with HED for the following policy changes: stricter and more intensive enforcement of breathalyzer, more prominent warning labels on products displaying the alcohol concentration and reduced opening hours.

**Table 4:** Test of association between HH where heavy episodic drinking (HED) occurs versus HH where no HED occurs and willingness to support national alcohol campaigns

National Campaign (Willingness to support)	No Heavy Episodic Drinking in HH (%)	Heavy Episodic Drinking in HH (%)	*P* value
Set the legal age for drinking at 21 years	997 (84.9)	514 (72.2)	0.006
Restricting alcohol advertisements on TV/radio/newspapers/cinema	869 (73.6)	368 (71.6)	0.215
Ban of all alcohol advertisements on TV/radio/newspapers/cinema	627 (53.1.)	267 (51.9)	0.351
Ban of all alcohol advertisements at cultural or sporting events	753 (63.8)	335 (65.2)	0.308
Delinking the consumption of alcohol with social or sexual success	724 (61.3)	326 (63.4)	0.220
Delinking the consumption of alcohol with driving or physical performance	814 (68.9)	361 (70.2)	0.316
Ban of radio stations playing songs which reference alcohol use	825 (69.9)	364 (70.8)	0.368
Stricter and more intensive enforcement of breathalyzer	1047 (88.7)	472 (91.8)	0.028
Holding sellers of alcohol responsible for the amount of alcohol they sell to patrons.	931 (78.8)	403 (78.4)	0.445
Advocating for proof of age to be shown before alcohol is sold to a buyer	1029 (87.1)	452 (87.9)	0.354
More prominent warning labels on products displaying the alcohol concentration	1008 (85.4)	463 (90.1)	0.005
More prominent warning labels on products displaying the harmful effects of alcohol	1032 (87.4)	462 (89.9)	0.082
Increased taxation on alcohol	1024 (86.7)	448 (87.2)	0.433
Increase the public education campaigns on responsible alcohol use in all settings including in schools	1070 (90.7)	463 (90.1)	0.380
Increased fines for drunk driving	1017 (86.3)	449 (87.4)	0.314
Reduced opening hours	485 (41.1)	153 (29.8)	0.001
Annoyed by the number of bars in your community	145 (12.3)	37 (7.2)	0.001
HH would like to see fewer bars operating in your community	189 (16.0)	65 (12.7)	0.009
HH disturbed by the noise coming from bars	94 (8.0)	21 (4.1)	0.011
HH disturbed by the patrons coming from the bars	96 (8.1)	23 (4.5)	0.004

It was also noted there was less acceptance of the following statements by HH where HED occurred: that the HH was annoyed by the number of bars in their community, that HH would like to see fewer bars operating in their community, that HH were disturbed by the noise coming from bars, or that HH were disturbed by the patrons coming from the bars.

## Discussion

This survey of a large cross-section of the population provides information to policy makers, civil society and public health institutions for addressing change in alcohol policies, laws and regulations in Trinidad and Tobago.

### How does these results compare with public opinion on alcohol internationally?

Recent international reviews suggest that public support is higher for the less effective interventions [18], for *example ‘support is lower for policies which seek to restrict the physical and economic availability of alcohol to the wider public..... and higher for policies directed towards informing, educating and treating targeted individuals’* [19]. In Australia several studies between 1998 and 2007, reported that 28–40% of respondents supported reducing trading hours or reducing outlet density where alcohol is served and more than 80% support stricter enforcement of laws and 69% supported increased health warnings on packaging [19]. Similar elements were found in South Africa [20].

Interestingly some elements of this pattern was found in this current study. This current study found that only 38% would support reducing trading hours or reducing outlet density. Similarly, in our study, over 90% would support stricter enforcement of the breathalyzer laws and more extensive labeling highlighting harmful effects. A similar finding for advertising is noted below. Where this study differed is in the support for taxation, whilst 80% of Trinidadian and Tobagonian respondents supported this, in Australia only 38–42% did so [19]. A similar low level of support for increased prices (34–58%) was found in South Africa [20].

### Why is support for these regulations so strong?

It is not easy to explain the findings in T&T compared to the rest of the world as this is the first study to examine the population’s willingness to support new policies and regulations on alcohol. Over the years this community has seen an increase in violent crimes, many linked to drug and alcohol use. Also many HH are affected by intimate partner violence and the effects of alcohol abuse [17]. This idea of second-hand drinking has support in the literature, in this case persons who experienced family or personal aggressive harms or who were concerned about a relative’s drinking were more supportive of restrictive alcohol policies [21]. These factors could be affecting the results we obtained. Most persons receive information about alcohol through school, employment, religious forums, media, including online, and peer interaction. Future qualitative type studies might be the mechanism to explore these factors.

### Outlet density and availability of alcohol

In our study, there was poor support for restrictions to be placed on the availability and accessibility to alcohol that would decrease the number of bars and rum shops in the neighbourhood or reduce the duration of their opening hours. The lack of support in this area did not significantly differ between HH that drank alcohol and those that did not. Internationally however, there is clear evidence that ‘*substantial changes in the number of alcohol outlets results in significant changes to alcohol consumption and related harms’* [1]. Further research needs to be done to better understand the reasons for these local findings.

### Advertising

The lack of policies on advertising and marketing in the T&T is an obvious gap. There are opportunities for interventions and this report suggested that there was public support. This survey found that many supported cautionary labels to be placed on alcoholic drinks and more than 50% supported delinking alcohol advertising with success, sex, driving and performance. More than half of all HH will support a policy banning alcohol advertisements at sporting events or restricting of advertisements in the media. As seen in this study significantly more non-alcohol consuming HHs (61%) was willing to support a ban on all advertising on TV compared to alcohol consuming HHs (48%). And half will support banning of all alcohol advertisements in the media. This is an important finding since ‘*Longitudinal studies consistently suggest that exposure to media and commercial communications on alcohol is associated with the likelihood that adolescents will start to drink alcohol, and with increased drinking amongst baseline drinkers’* [22]. There is currently a call for banning alcohol advertisements by the Global Alcohol Policy Alliance [23]. International public opinion polls suggest that roughly similar proportions of other populations support such marketing restrictions [20]. In Australia between 40 and 70% supported advertising being reduced or banned [19]. In the US, one study reports that 60% support alcohol advertising and promotion restrictions [24]. Similarly only half of this population, support banning advertising of alcohol.

### Adolescents

The results show that there was good support for more rigorous control of young people drinking. When asked about the implementation of access controls concerning young people (age < 21 years) support was greater, with more than 3/4 of respondents supporting setting the legal age where alcohol consumption is allowed to 21 years. It may be that young people are viewed as having diminished responsibility and therefore restrictions to access alcohol for this age group is warranted. The international literature supports the view that ‘*minimum legal purchase age is effective in reducing road fatalities and other harms with minimal enforcement, but enforcement substantially increases effectiveness and the cost’* [1]. Recent reports suggests that in South Africa [19] and South Korea [25] there is similar support for increasing the alcohol purchasing age to 20–21 years. Focusing attention on regulations pertaining to adolescent purchasing or possession of alcohol may be an effective use of resources. Increasing the age of alcohol purchasing to 21 in the USA has been accredited with saving the lives of 21,000 persons between 1975 and 2002 [26].

### Taxation

The majority of HH will support increased taxation on alcohol. This is a promising intervention for many governments, increasing revenue while reducing consumption and harm. It also has the potential to reduce consumption among young people who may have less disposable income.*“Studies have consistently demonstrated that alcohol prices have an effect on levels of consumption and related harms, including mortality rates, crime and traffic accidents.”* [1].

### Study strengths

The survey had a large sample size and a high response rate. Although this high response rate is in part due to the experience of the interviewers and the relatively short survey instrument, participants also showed keen interest in the area of study “alcohol”. Many HHs congratulated the interviewers for investigating alcohol and several persons volunteered to participate in a follow-up study of their personal and family experience of alcohol. With the exception of a ban on all TV advertising, the majority of HHs in this survey were willing to support changes in policy, independent of alcohol consumption status. This suggests that the population is receptive to the dissemination of information pertaining to alcohol and to the implementation of regulations, laws and policies that have been demonstrated in other countries to be effecting change in alcohol use or misuse. Multiple internet searches using the terms: alcohol, public, opinion and the names of various Caribbean nations gave no relevant papers, so this paper may represent the first such attempt to capture a Caribbean population’s opinion on this issue.

### Limitations

It is important to state that while our validation process of the instrument ensured content, face and cultural validity, this scale has not been used or tested before.

Several areas of interest were not studied because of space limitations in questionnaire and lack of information when developing the instrument, these include driving restrictions for drunk drivers, Selling alcohol at gas stations, Alcohol marketing on the internet and social media. Additionally our sample size limited by funding and the full number of EDs could not be surveyed. Although the response rate was high overall, one of the selected enumeration districts was in a very high income neighbourhood, there was limited access to the HH, many with high walls and security. Only a few responses was obtained. Another limitation was not asking whether at least one family member or close relative was involved in a serious or fatal accident, or other negative experience, in which drinking was involved. Such an experience might have influenced their choice of restrictions.

Care should be taken in the interpretation of the items in the chi-square analysis where *p* < 0.05, as closer study of the OR show many approaching or just under an OR of 1 or no effect. Only one item, ‘banning TV advertisements’ was significantly supported by HH not using alcohol.

## Conclusions

Apart from restrictions in density of outlets and reduction in opening times for alcohol outlets the majority of HHs in T&T are willing to support changes in policies around alcohol, including many of the policies shown by the WHO to be effective in reducing the harmful consumption of alcohol. The long established alcohol industry has considerable support among governments and other sectors within the region and the change suggested in this paper will be difficult. This will require all stakeholders to have an input, including Civil Society Organizations(CSO). There are roles for a CSO such as the Healthy Caribbean Coalition and the Caribbean Public Health Agency (CARPHA) in promoting this agenda.

## Abbreviations

CCH II: Caribbean Cooperation in Health II
CSO: Central Statistical Office
CSSP: Continuous Sample Survey of the Population
DALY: Disability Adjusted Life Years
ED: Enumeration District
ESC: English-speaking Caribbean
HED: Heavy Episodic Drinking
HH: Household
LAC: Latin America and The Caribbean
NASHTT: National Alcohol Survey of Households in Trinidad and Tobago
NCD: Non Communicable Disease
SPSS: Statistical Package for Social Sciences
T&T: Trinidad and Tobago
WHO: World Health Organization
